# DeepLuAd: Semantic-guided virtual histopathology of lung adenocarcinoma via stimulated Raman scattering

**DOI:** 10.7150/thno.125443

**Published:** 2026-01-01

**Authors:** Liyang Ma, Yuheng Guo, Min Du, Yongjun Cai, Yingjie He, Qingjun Meng, Zhijie Liu, Yichuan Lan, Ming Li, Minbiao Ji, Lin Qi

**Affiliations:** 1 Huadong Hospital Affiliated to Fudan University, Department of Radiology, 200040 Shanghai, China.; 2 State Key Laboratory of Surface Physics and Department of Physics, Shanghai Key Laboratory of Metasurfaces for Light Manipulation, Endoscopy Center and Endoscopy Research Institute, Zhongshan Hospital, Fudan University, 200433 Shanghai, China.; 3 Huadong Hospital Affiliated to Fudan University, Department of Pathology, 200040 Shanghai, China.

**Keywords:** lung adenocarcinoma, label-free imaging, stimulated Raman scattering, deep learning, tissue histopathology

## Abstract

**Rationale:** Accurate histologic grading of lung adenocarcinoma is essential for guiding clinical management. Conventional hematoxylin and eosin (H&E) staining provides morphological information but lacks biochemical specificity, limiting quantitative analysis of tissue subtypes within the heterogeneous lung cancer microenvironments.

**Methods:** We developed DeepLuAd, an AI-powered platform integrating label-free stimulated Raman scattering (SRS) microscopy with semantic-guided deep learning. The platform enables automated tumor grading, segmentation, cellular-level morpho-chemical quantification, and unsupervised virtual H&E staining.

**Results:** DeepLuAd achieved a mean intersection-over-union (mIoU) of 80.43% across major lung tissue subtypes and reached a grading concordance rate of 76.2% with pathologist diagnoses (16/21 cases). The approach also enabled quantitative mapping of lipid-to-protein ratio heterogeneity within tumor and stromal compartments, revealing biochemical signatures of disease progression.

**Conclusions:** DeepLuAd provides an interpretable and scalable framework for digital lung adenocarcinoma analysis, unifying morphological and biochemical information without the need for staining. The method demonstrates potential for broader application to other solid tumors in AI-enhanced histopathology.

## Introduction

Lung cancer remains one of the most prevalent and deadliest malignancies worldwide, with lung adenocarcinoma accounting for approximately 45.6% of male and 59.7% of female cases globally 1. Rapid and accurate diagnostic grading of lung adenocarcinoma is crucial for improving patient outcomes. According to the pathological grading guidelines established by the International Association for the Study of Lung Cancer (IASLC), tumor grading is based on whether the combined area of primary dominant and high-grade patterns exceeds 20%, a threshold that significantly impacts prognosis and survival outcomes 2, 3. However, traditional hematoxylin and eosin (H&E) staining - the gold standard for tissue histopathology, mainly provides morphological information and heavily relies on pathologists' subjective estimation. Hence quantitative evaluation of the area percentage of each histologic grade is highly challenging, particularly for heterogeneous tumor types 4-7. As a result, conventional histopathology faces limitations in diagnostic efficiency, accuracy, and clinical reliability, highlighting a growing need for automated, high-precision analysis systems tailored for lung adenocarcinoma diagnosis.

Stimulated Raman scattering (SRS) microscopy has become a unique label-free imaging technique, offering rapid and chemical-specific visualization of endogenous molecules based on their vibrational signatures, demonstrating broad research applications in biology, medicine, and material sciences 8-18. SRS has shown success in stain-free histopathology, achieving near real-time, high-precision diagnosis of various disease tissues, including brain tumor, gastric cancer, breast cancer, prostate cancer, and etc. 19-32. Using unprocessed fresh tissues, SRS was able to conduct timely diagnosis on surgical tissues, endoscopic biopsies, and needle biopsies 33-36. In addition to SRS, other nonlinear optical signals, such as second harmonic generation (SHG), could be simultaneously harvested to specifically visualize collagen fibers - an important component of extracellular matrix 37, 38. The combined modalities were able to capture both cellular and stromal components without staining 33, 34, 39-43. Despite previous efforts in tumor histology, applications of SRS in lung adenocarcinoma remain limited, largely due to the complexities of lung tissues including morphological and biochemical heterogeneities. In particular, precise tumor grading of lung adenocarcinoma is essential for risk stratification and treatment management, yet it remains challenging for conventional SRS imaging and analysis.

Parallel to advances in optical techniques, artificial intelligence (AI) has revolutionized research in medical imaging and histopathology 44, 45. While the integration of AI models with SRS has enabled notable breakthroughs in diagnostic classification, segmentation and virtual staining 24, 27, 28, 33, 34, 39, 46, 47, previous approaches predominantly rely on patch-level convolutional neural networks (CNNs) such as U-Net, which struggle to resolve intricate histoarchitectural features of lung tissues where pixel-level segmentation is essential for precise analysis. Therefore, several key challenges remain in the current SRS-AI frameworks: (1) Lack of pixel-level precision to delineate fine morphological boundaries for accurate subtype identification; (2) Absence of semantic correspondence to associate biochemical distributions with histologic features; (3) Limitations in virtual staining for complex tissue histoarchitectures to achieve simultaneous structural fidelity and biochemical consistency.

These challenges underscore an urgent need for more advanced frameworks capable of capturing pixel-level histological semantics and biochemical heterogeneity to achieve interpretable, label-free histopathology. Here we present DeepLuAd, a multi-task AI-platform that performs histological segmentation, biochemical quantification, and virtual staining on SRS images of lung adenocarcinoma. The integrated framework enables label-free, pixel-level segmentation of major lung adenocarcinoma subtypes, which subsequently facilitates objective pathological grading into three prognostic stages. It delivers cellular-level morphological and biochemical profiling based on lipid and protein distributions, and provides comprehensive insights into the complex tumor microenvironment. The modular and scalable design of DeepLuAd can be readily extended to diverse diseases contexts, offering a versatile tool for intelligent tissue analysis in digital pathology and precision diagnosis.

## Results

### Label-free microscopy platform and workflow for lung tissue imaging

**Figure 1:**
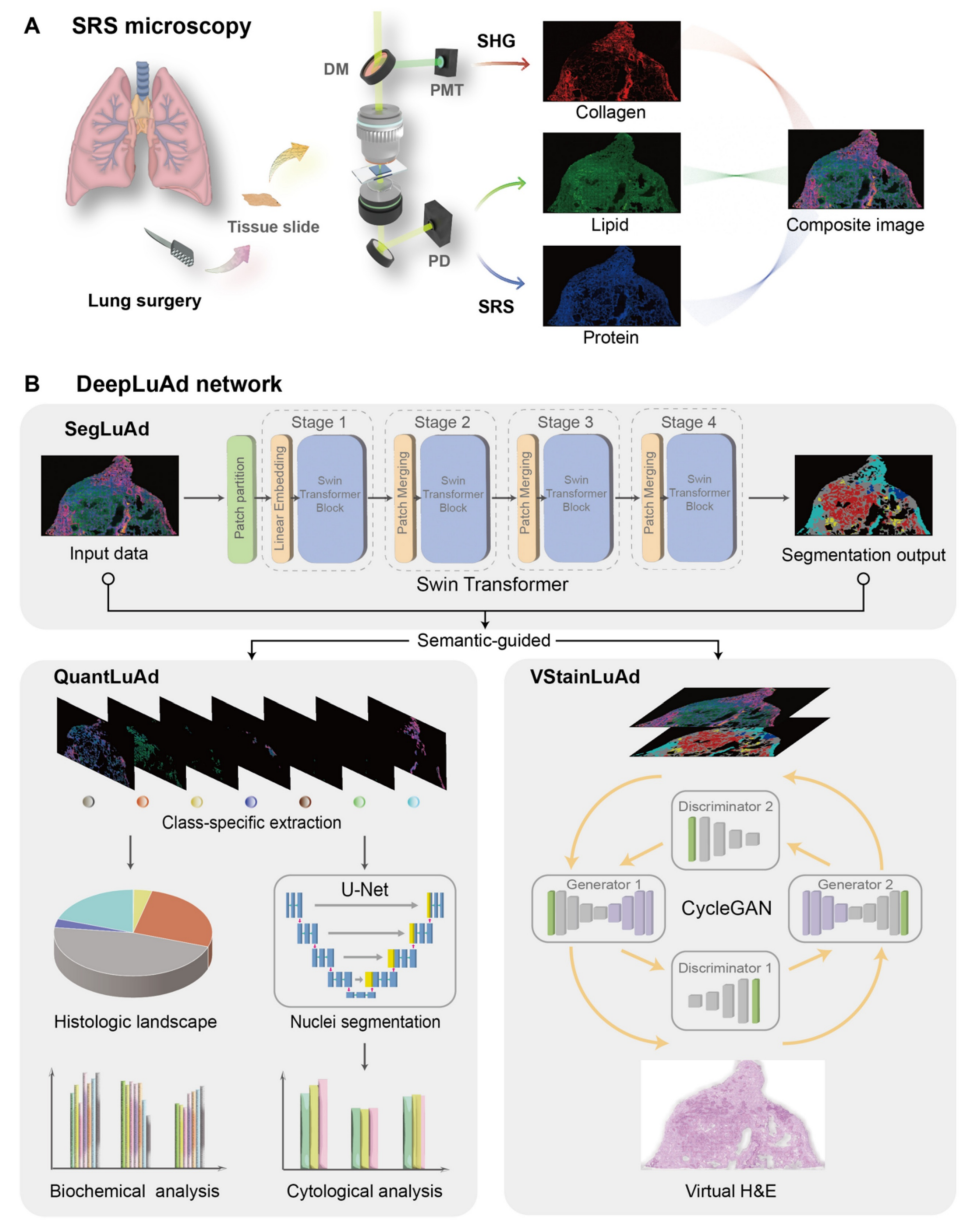
** Workflow of SRS imaging and deep-learning platform for virtual histopathology of lung adenocarcinoma.** (A) Label-free SRS microscopy for lung surgical tissue specimens, with dual SRS channels of lipid (green) and protein (blue), and an SHG channel of collagen fibers (red). (B) Multi-task DeepLuAd network composed of three AI modules: SegLuAd for semantic segmentation of tissue classes; QuantLuAd for quantitative biochemical and cytological feature analysis; and VStainLuAd for virtual H&E staining.

A multimodal nonlinear optical microscopy system based on SRS and SHG was constructed to achieve high-resolution, label-free visualization of lung tissue histology. The detailed setup of the optical system is shown in Figure S1 and described in the Methods. In our imaging system, SRS microscopy acquired dual-channel bond-selective vibrational signals at 2845 cm⁻¹ (CH₂) and 2930 cm⁻¹ (CH₃), which were subsequently decomposed into lipid and protein distributions using spectral unmixing algorithms 27, 48, 49. Meanwhile, SHG signal was collected to represent collagen fibers in the bronchial structures and extracellular matrix. The two unmixed SRS images together with the SHG channel offered the primary chemical contrast for histological analysis. The final composite images were RGB color-coded: red for collagen, green for lipid and blue for protein (Figure 1A). These SRS/SHG images provided the main data in our work, serving as training and test dataset for the subsequent deep-learning models.

The overview of the integrated AI platform - DeepLuAd is illustrated in Figure 1B, which contains three sub-modules responsible for segmentation, quantification and virtual H&E staining. SegLuAd performs pixel-level segmentation of lung tissue from SRS images, providing spatial context for downstream analysis. QuantLuAd leverages segmentation map to extract class-specific biochemical and cytological features, including lipid/protein composition and cellular morphology. VStainLuAd integrates structural priors from SegLuAd to generate virtual H&E images that resemble conventional histology. Together, the multi-task AI platform transforms raw SRS data into quantitative and interpretable histopathologic results.

### Revealing key histologic features of lung adenocarcinoma

**Figure 2:**
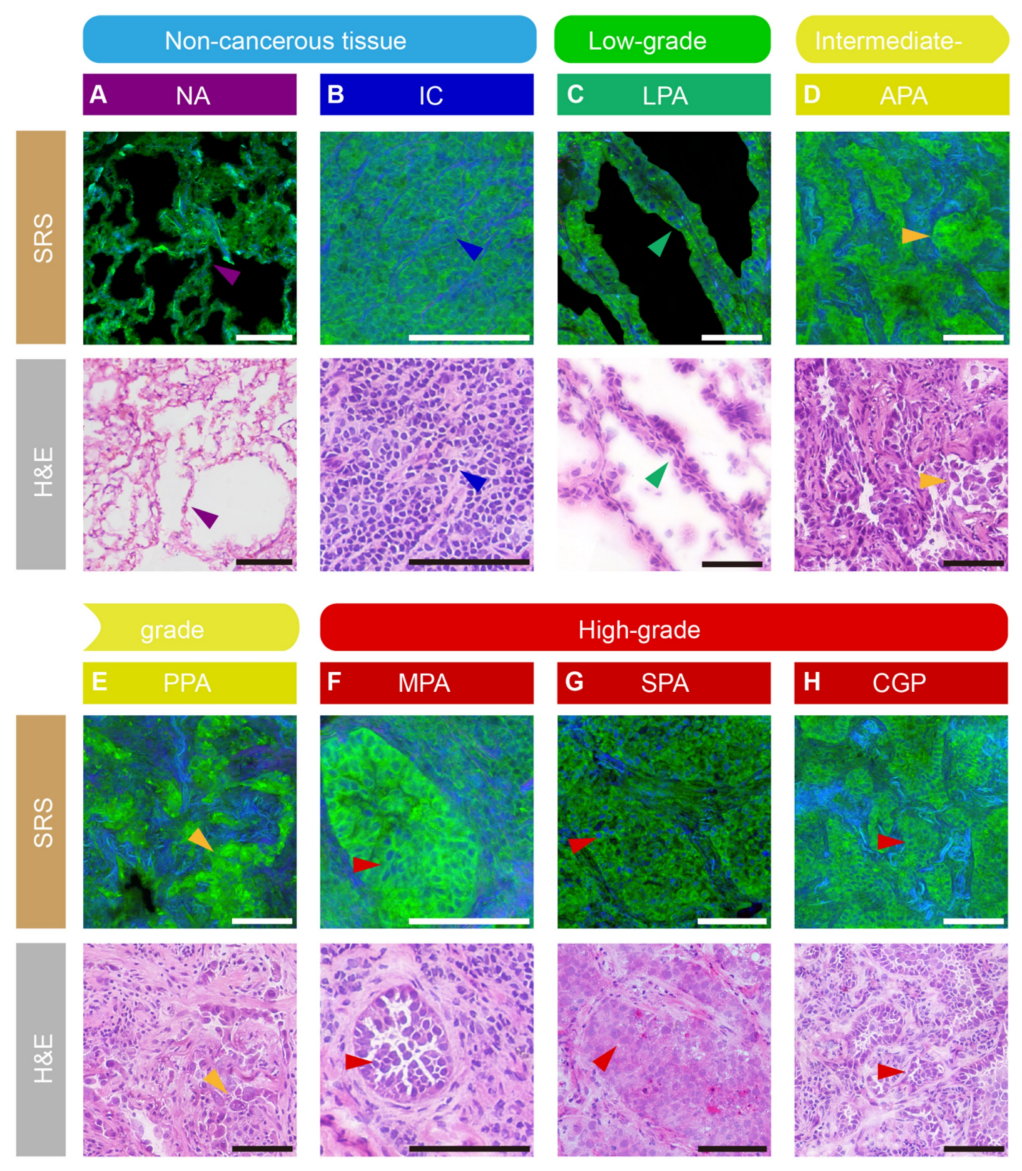
** Representative SRS and H&E images of adjacent sections reveal major histologic features of tissue subtypes.** Arrows indicate key diagnostic morphological cues. (A) Normal alveoli (NA). (B) Immune cells (IC). (C) Lepidic predominant adenocarcinoma (LPA). (D) Acinar predominant adenocarcinoma (APA). (E) Papillary predominant adenocarcinoma (PPA). (F) Micropapillary predominant adenocarcinoma (MPA). (G) Solid predominant adenocarcinoma (SPA). (H) Complex glandular patterns (CGP). Scale bars: 100 μm.

We then assessed the ability of SRS microscopy to visualize critical histopathologic features of lung adenocarcinoma subtypes. Unlike other types of tissues, lung tissues include rich cavity structures that are easily deformed under external force, making it challenging to directly image on fresh lung tissues without perturbing the native morphologies. Therefore, in this study, we chose to work on thin frozen tissue sections. For histologic verification, a pair of adjacent sister sections were created, one of which was sent for conventional H&E staining, and the other was imaged under SRS microscope. Although minor morphological variations may occur between adjacent sections due to tissue processing and sectioning—such as slight differences in tissue folding, localized cellular packing, or stromal thickness—the major morphological characteristics that determine tissue identity, including the overall glandular architecture, pattern-level stromal organization, and grade-associated trends in nuclear morphology and cell density, remain well preserved. Figure 2 shows representative SRS and corresponding H&E images from adjacent tissue sections, demonstrating that SRS effectively differentiates histoarchitectural features across tissue subtypes, achieving diagnostic capability comparable to conventional histopathology. For clarity, arrows mark representative morphological features that guide subtype recognition. Non-cancerous tissues reveal characteristic structures: normal alveolar (NA) exhibits gas-exchange sacs lined by a single layer of epithelial cells (Figure 2A), while immune cells (IC) appear as densely packed populations with high nuclear-to-cytoplasmic ratios (Figure 2B).

Among malignant subtypes, low-grade adenocarcinoma is mainly represented by lepidic predominant adenocarcinoma (LPA), maintains alveolar architecture with tumor cells spreading along alveolar walls (Figure 2C). Intermediate-grade lesions include: acinar predominant adenocarcinoma (APA) displaying well-formed, round/oval glandular lumina (Figure 2D); and papillary predominant adenocarcinoma (PPA) with fibrovascular cores lined by neoplastic epithelial cells, forming papillary projections (Figure 2E). High-grade subtypes comprise: micropapillary predominant adenocarcinoma (MPA) showing alveolar-space tumor clusters without fibrovascular cores (Figure 2F); solid predominant adenocarcinoma (SPA) featuring sheets minimally differentiated tumor cells (Figure 2G); and complex glandular patterns (CGP) exhibiting cribriform/fused glands (Figure 2H). The three high-grade histologic features correlate with metastatic propensity and adverse prognosis. The interpretation of these morphological patterns follows the diagnostic criteria defined in the IASLC lung adenocarcinoma classification 2, and all regional assignments were jointly confirmed by two experienced pulmonary pathologists. These results demonstrate that SRS imaging preserves the essential morphological features needed for reliable subtype recognition.

Lipid/protein based chemical contrast plays a key role in the identification of various histologic features. Cell nuclei exhibit strong protein-dominated signals due to high protein density, while the cytoplasm is relatively enriched in lipid signals originating from intracellular lipid droplets and lipid-rich organelles. The alveolar wall consists of collagen fibers with detectable SHG signals in stromal regions, but shows minimal response in the thin epithelial lining of normal alveoli. In contrast, tumor cells display elevated lipid and protein content with altered distributions, reflecting metabolic reprogramming and dense cellular packing.

### Deep learning-based segmentation and grading of lung adenocarcinoma

**Figure 3:**
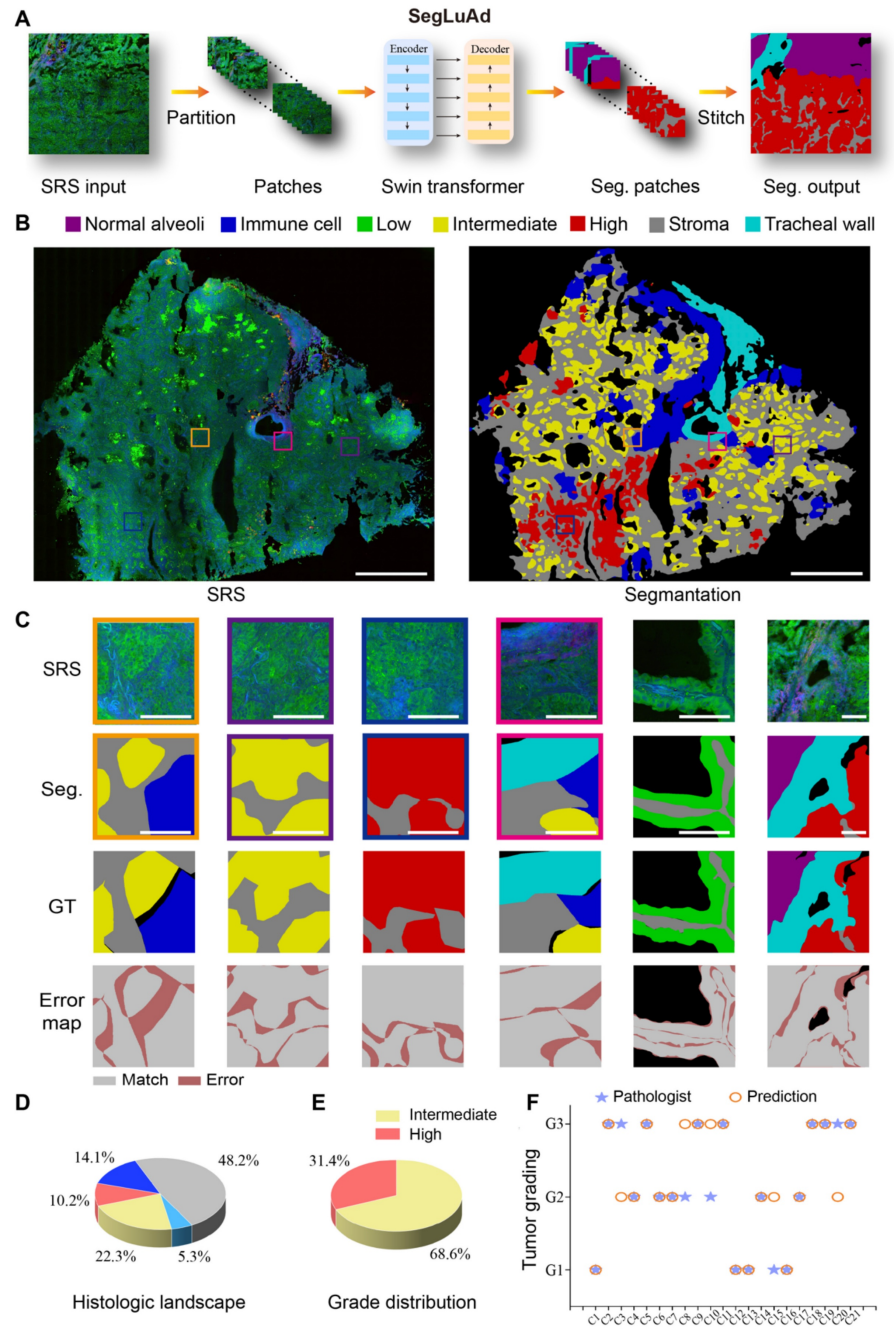
** SegLuAd module for histologic segmentation of seven tissue classes.** (A) Schematic workflow of SegLuAd pipeline. (B) Representative whole-slide SRS image and corresponding segmentation output. (C) Magnified images of representative regions: SRS data, segmentation results, annotated ground truth and error map. (D) Quantified area percentages of different tissue classes. (E) Tumor grade distribution derived from subtypes partition. (F) Clinical validation on an external cohort of 21 cases, showing a grading consistency rate of 76.2%. Color coding for (B-D): Low grade (green), Intermediate grade (yellow), High grade (red), Immune cells (dark blue), Normal alveoli (purple), Stroma (gray), Tracheal wall (cyan). Scale bars: 1000 µm in (B), 100 µm in (C).

Accurate delineation of histological subtypes in lung adenocarcinoma is critical for reliable pathological grading and prognostic stratification. However, conventional image-level classification approaches lack spatial resolution for precise pathological analysis on heterogeneous tissue sections. As the first module of DeepLuAd, SegLuAd is developed for automated segmentation of seven tissue classes on SRS images (Figure 3A), including normal alveoli, immune cells, low-grade, intermediate-grade, and high-grade adenocarcinomas, stroma, and tracheal wall.

SegLuAd is built upon the Swin Transformer backbone, incorporating domain-specific optimizations tailored for high-resolution SRS images. Specifically, the original large-area SRS images were first cropped into non-overlapping 2048 × 2048-pixel patches to facilitate training. Each patch was then standardized, resized to 600 × 600 pixels, and randomly cropped to 512 × 512 pixels before feeding into the network. These preprocessing steps were designed to enhance training stability, ensure consistent input size, and preserve critical tissue boundary information.

Our dataset consisted exclusively of SRS images. Corresponding H&E-stained frozen sections were used as references by experienced pathologists to assist in the annotation of various subtypes. Pathologists manually delineated regions of interest at the pixel level, categorizing tissues into seven predefined tissue classes mentioned earlier. A total of 80 annotated SRS slides from 50 patients were used for model development. These were randomly split into a training set and validation set in a 3:1 ratio, with segmentation optimized using a combination of Dice Loss and Cross-Entropy Loss. Details of the patient cohort and annotation distribution are provided in Figure S2. An additional independent test cohort of 21 cases was used for external validation, as described in a later section. The complete workflow of SegLuAd training and the case-level histological grading strategy is illustrated in Figure S3, outlining image preprocessing, dataset construction, model training with cross-validation, and the subsequent area-based rules used to assign tumor grades. After iterative training, SegLuAd demonstrated strong segmentation performance across tissue types. As summarized in Table 1, the Intersection over Union (IoU) scores for normal alveoli, immune cells, low-grade, intermediate-grade, high-grade, stroma, tracheal wall, and background were 84.06%, 70.97%, 79.23%, 74.64%, 85.16%, 87.94%, 82.93%, and 78.47%, respectively. To provide a more comprehensive evaluation of segmentation performance, additional metrics including pixel accuracy (Acc), Dice coefficient, precision, recall, and boundary F1-score (BF1) were also assessed for each tissue class. On average, SegLuAd achieved a mean IoU of 80.42% and a mean precision of 92.96%, together with mean Dice, accuracy, recall, and boundary F1-scores of 89.05%, 97.59%, 85.56%, and 73.94%, respectively. These results indicate stable performance across diverse lung adenocarcinoma subtypes and stromal components. The definitions of these metrics are provided in the Methods section.

To further assess the performance of our model, we compared SegLuAd with several widely used segmentation networks, including PSPNet, HRNet, DeepLabV3, U-Net, and Vision Transformer (ViT) (as shown in Supplementary Table S1). SegLuAd consistently outperformed all conventional CNN-based models in both mIoU and pixel accuracy, and achieved comparable or superior performance relative to ViT while maintaining lower computational complexity. Moreover, the customized variant SegLuAd demonstrated further improvements over the baseline, achieving the highest segmentation performance across all evaluated models.

To illustrate the model behavior and subtype differentiation, we visualized representative segmentation results on SRS validation dataset. As shown in Figures 3B and 3C, the network efficiently produced segmentation outputs that closely matched the annotations by pathologist, accurately distinguishing tumor from stroma and normal alveolar walls, and correctly identifying low-, intermediate-, and high-grade tumor regions. The full-section error map, highlighting matched and mismatched regions between prediction and ground truth, is shown in Figure S4. Most mismatches are located along tissue boundaries, reflecting natural histologic transitions rather than subtype-defining differences, and thus exert only minimal influence on diagnostic interpretation and grading. Importantly, the segmentation results preserved the integrity of glandular and solid architectures while accurately separating additional components such as immune cell regions and tracheal wall, indicating robust performance in capturing clinically relevant structural organization. Building on these results, the area percentage of each tissue type could be quantitatively extracted from the segmentation maps, enabling precise tumor grading for patient cases according to the IASLC clinical criteria 2: cases with ≥ 20% high-grade patterns are classified as Grade 3; otherwise, the tumor is graded according to the predominant histological subtype; i.e., low-grade predominance (lepidic) is classified as Grade 1; intermediate-grade predominance (acinar or papillary) is classified as Grade 2. In an example case, the segmentation results showed the partition of all tissue types (Figure 3D), including 48.2% of stroma, 10.2% of high-grade and 22.3% of intermediate-grade tumor. These could be converted to the percentages of tumor subtypes among the total area of tumor: 31.4% of high-grade and 68.6% of intermediate-grade (Figure 3E), resulting in the final tumor grade of 3 according to the criteria. To further demonstrate the grading workflow, another representative case (Figure S5) shows 65.5% intermediate-grade and 10.3% low-grade tumor on the whole slide; after normalization within tumor regions, the distribution consists of 86.4% intermediate-grade and 13.6% low-grade pattern, with the high-grade component below the 20% threshold, resulting in a final grade of 2.

To further evaluate the clinical relevance of our approach, we compared the AI-predicted tumor grades with consensus pathology reports determined by two experienced pathologists across 21 independent cases (Figure 3F, Table S2). Our model achieved a grading consistency rate of 76.2% (16/21 cases), demonstrating the potential of SegLuAd for automated lung adenocarcinoma grading via label-free tissue histology.

### Subtype-resolved biochemical and cytological profiling

One major advantage of SRS is the capability of quantitative chemical analysis exploiting the linear relationship between signal intensity and molecular concentration. Previous chemical analysis of tissues were usually conducted on the image-basis, averaging target chemical signals over the whole-slide images or certain image areas, lacking spatial resolution for each tissue subtype. Tissue-type-specific analysis is challenging for highly heterogeneous tissue like lung, where different tissue types interweave together (Figure 3). To enable subtype-resolved biochemical quantification, we developed an AI module (QuantLuAd) that integrates semantic-segmentation with pixel-level chemical and cytological analysis. The module automatically extracts key parameters from spectrally decomposed SRS images comprising the intensity maps of lipid, protein and collagen. These parameters include lipid intensity, protein intensity, lipid-to-protein ratio (L/P), nuclear-to-cytoplasm ratio (N/C), average cell size, and cell density. The multi-component SRS images combined with segmentation masks were utilized to analyze region-specific biochemical distributions and morphologic features.

**Figure 4:**
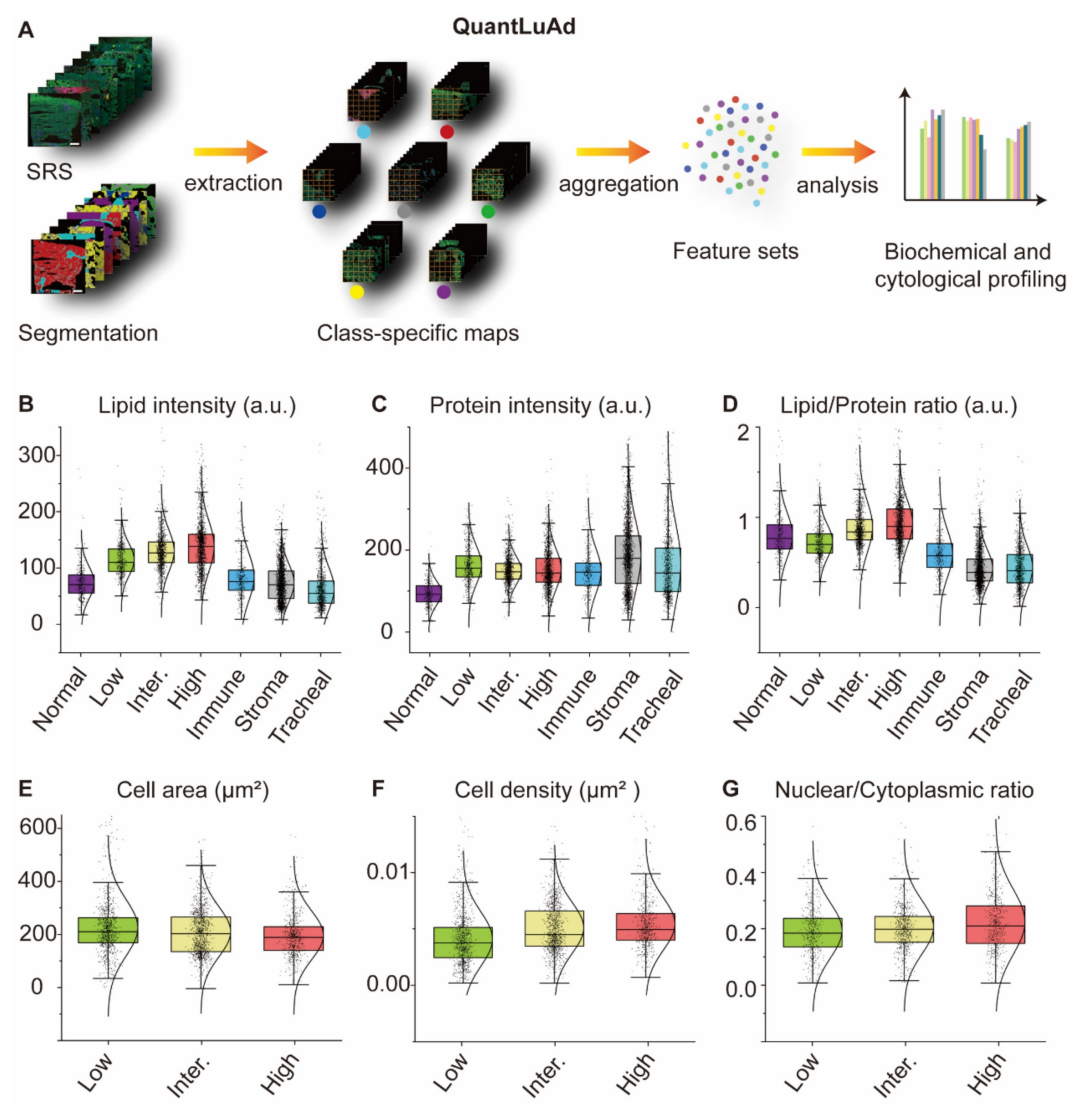
** QuantLuAd module for biochemical and cytological analysis based on segmentation results.** (A) Workflow of QuantLuAd, including segmentation-based image extraction, aggregation and statistical analysis. (B-D) Biochemical profiling including the intensity distributions of lipid, protein and lipid/protein ratio across the seven tissue subtypes. (E-G) Cytological profiling including cell size, cell density and nuclear/cytoplasmic ratio in low-, intermediate-, and high-grade tumor regions. a.u.: arbitrary units. Scale bar: 1 mm in (A).

We adopted 20 representative image slides to evaluate the model performance across all tissue types and tumor grades. To avoid sampling bias due to tissue size variation, the whole-slide images were sliced into tiles with 512 × 512 pixels, each of which was treated as an analytical unit, and ~1,000 representative patches were randomly sampled from each tissue type for statistical analysis (Figure. 4A).

The statistical results for all the parameters are summarized in Figures 4B-4G. In detail, the mean lipid intensity appeared relatively low in normal alveoli (70.2 a.u.), in contrast to the increasing values in low-grade, medium-grade, and high-grade subtypes, with values of 109.8 a.u., 127.2 a.u., and 138.3 a.u., respectively (Figure 4B). This progressive increase likely reflects lipid metabolic reprogramming during tumor progression, with accumulated lipids serving both as membrane components and energy substrates to sustain rapid cancer cell proliferation 50-53. Compared to cancer regions, lipid content in immune cells, stroma, and tracheal walls exhibited lower values (~70 a.u.). Regarding the mean protein intensity, except that normal alveoli demonstrated low protein level (~90 a.u.), the rest six tissue types showed significantly higher protein content ranging from 140 a.u. to 180 a.u. (Figure 4C). This may correlate with increased cellular density and cytoplasmic complexity in tumor and stromal regions, supporting the biosynthetic demands of cancer cells 54. As for lipid-to-protein ratio, an increasing trend from low-grade to high-grade subtypes was observed (Figure 4D), agreeing with increased lipid contents in higher grade tumors. In immune cells, stroma, and tracheal walls, L/P ratios appeared remarkably lower than those of cancerous tissues.

To enable subtype-specific cytological analysis, we trained a U-Net model to identify and delineate individual cell nuclei in SRS images, enabling the quantification of cell nuclear population, average cell size, nuclear-to-cytoplasm ratio, and cell density across different tumor subtypes. The model achieved IoU = 74.1%, Dice = 85.1%, Precision = 87.5%, Recall = 82.9%, and Accuracy = 91.3%, confirming robust and reliable nuclei segmentation for downstream quantitative analysis. Detailed workflow along with representative output example was shown in Figure S6. Statistical results revealed that average cell size gradually decreased from low-, intermediate-, to high-grade tumors (Figure 4E), with values of 221.5 μm², 212.8 μm², and 198.0 μm², respectively. This trend is consistent with the histological feature of cellular pleomorphism and reduced cytoplasmic volume in high-grade tumors 55. Average cell density in the alveolar space gradually increased (Figure 4F), reaching 0.0037 μm⁻², 0.0045 μm⁻², and 0.0049 μm⁻² from low to high grade, respectively. Nuclear-to-cytoplasm ratio also showed an increasing trend (Figure 4G), with average values of 0.18, 0.20, and 0.21 from low to high grade. These cytological features reflect classic hallmarks of malignancy, including nuclear enlargement and crowding, validating the biological relevance of the model-extracted parameters 55-57.

To further validate the robustness of SegLuAd-based quantitative analysis, we compared the analytical results derived from SegLuAd segmentation with those obtained from ground-truth (GT) pathologist annotations. As shown in Figure S7, the statistical measurements derived from GT segmentation exhibit the same subtype-dependent trends as those in Figure 4, indicating strong concordance between the two sources of segmentation. The agreement analysis in Table S3 demonstrates a Spearman correlation (SP) and concordance correlation coefficient (CCC) of approximately 0.99, confirming that SegLuAd preserves the critical structural information required for reliable downstream histologic quantification. In addition, case-level comparisons for low-, intermediate-, and high-grade subtypes (Table S4) further support the consistency between the two segmentation approaches, underscoring the reliability of SegLuAd for subtype-resolved morphochemical analysis.

These results indicated the accuracy and robustness of our analytical framework in quantifying biochemical signals (lipid intensity, protein intensity, L/P ratio) and cytological features (cell size, cell density, N/C ratio). Our model revealed class-specific morpho-chemical patterns in lung adenocarcinoma, providing new perspectives for pathological interpretation.

### Semantic-guided virtual H&E staining

Transforming label-free SRS images to standard H&E staining results is crucial for the direct interpretation by pathologists, as well as enhancing fine histologic features that appear obscure in original SRS images. While virtual staining algorithms have been applied to SRS in brain and gastrointestinal tissues 28, 58, the more histologically diverse structures of lung tissue — including alveolar cavities, collagen-rich stroma, and various tumor architectures — challenges the realization and generalization of effective virtual H&E for SRS-based lung histology, especially in the absence of paired image data. We developed VStainLuAd, by integrating an unsupervised framework based on CycleGAN (Cycle-Consistent Generative Adversarial Network) with structure-guided sementic priors from SegLuAd segmentation results, specifically tailored for the complex architecture of lung tissue.

**Figure 5:**
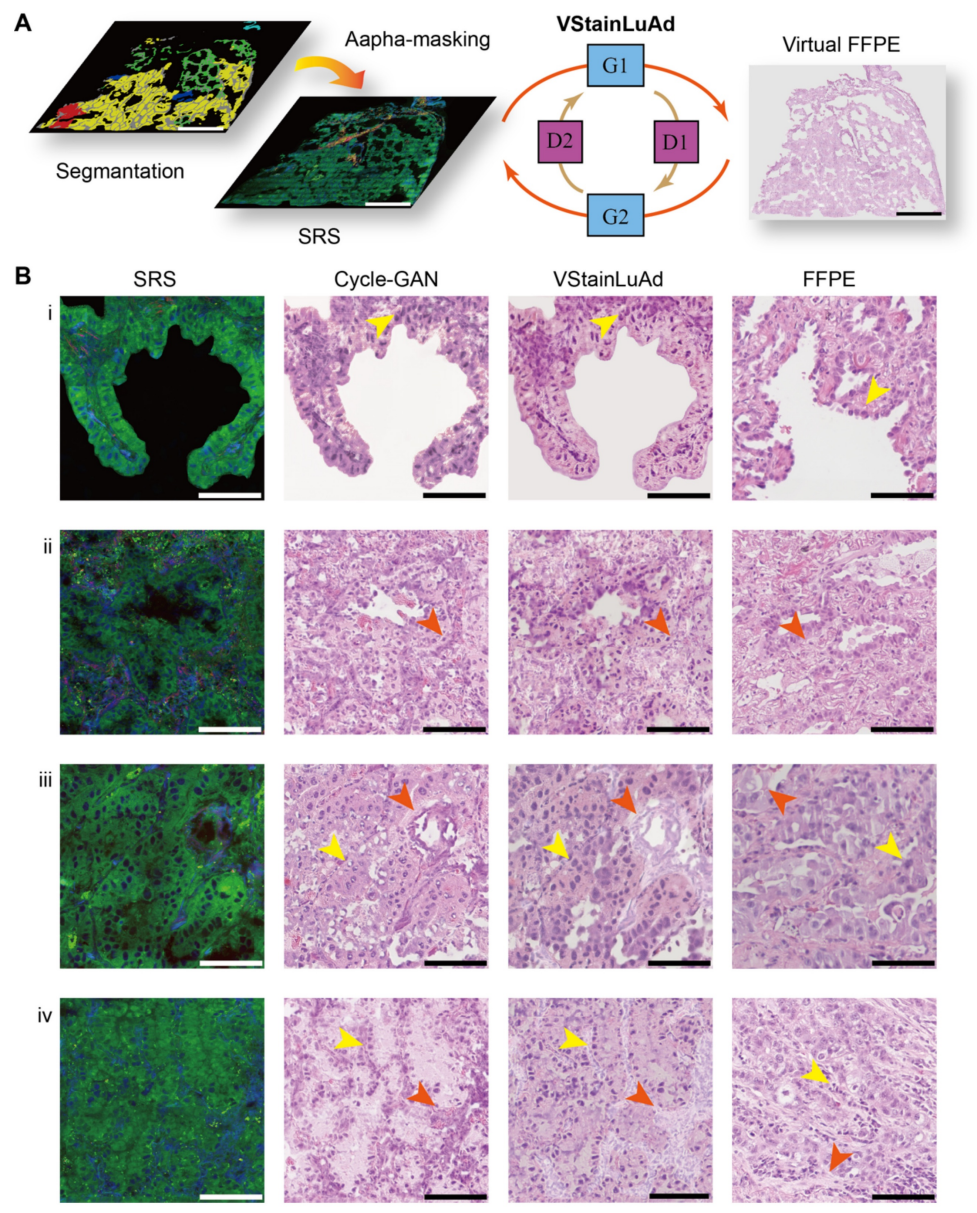
** Semantic-fused virtual staining with VStainLuAd module.** (A) Workflow of VStainLuAd framework incorporating segmentation maps into a CycleGAN architecture for virtual FFPE-style H&E staining. (B) Comparison of virtual staining results using conventional CycleGAN, VStainLuAd, and FFPE H&E stains (truth) across four representative lung tissue regions, showing improved nuclear-cytoplasmic contrast (yellow arrow) and enhanced structural fidelity in stromal (orange arrow). Scale bars: 1 mm in (A), 100 µm in (B).

As illustrated in Figure 5A, the VStainLuAd framework adopts a standard bidirectional CycleGAN architecture with two generators (G1, G2) and two discriminators (D1, D2), training on unpaired data of SRS and H&E. To enhance the network's structural perception and tissue-specific sensitivity, we introduced a semantic guidance mechanism. Specifically, the segmentation probability maps generated by our SegLuAd network were used to construct alpha masks, which were fused with the original SRS images and fed into the generator. By assigning different weights to distinct tissue types, this semantic regularization encouraged the network to focus on structurally relevant regions during training, thereby improving morphological reconstruction and reducing miscoloration.

We employed a two-stage training strategy (Figure S8). In the first stage, the model was pretrained on brain SRS images 28, which exhibit relatively regular structure and chemical distributions, allowing the network to learn stable base mappings from SRS signals to H&E-type color space. In the second stage, we adopted a transfer learning strategy, where the brain-SRS-pretrained model was fine-tuned on lung tissue data using semantic guidance, enhancing staining fidelity for complex lung histoarchitectures. Compared with conventional CycleGAN, and our semantic-guided VStainLuAd demonstrated virtual staining results with more accurate cytoplasmic and nuclear contrast, closely resembling the features in true formalin-fixed, paraffin-embedded (FFPE) sections (Figure 5B-i). Additionally, VStainLuAd effectively distinguished stromal and nuclear regions (Figure 5B-ii, iii), which typically present challenges for traditional CycleGAN. Particularly in high-grade adenocarcinoma regions (Figure 5B-iii, iv), VStainLuAd clearly revealed dense cellular arrangements and deeply stained nuclei, whereas traditional CycleGAN exhibited notable structural blurring and inaccurate nuclear positioning.

**Figure 6:**
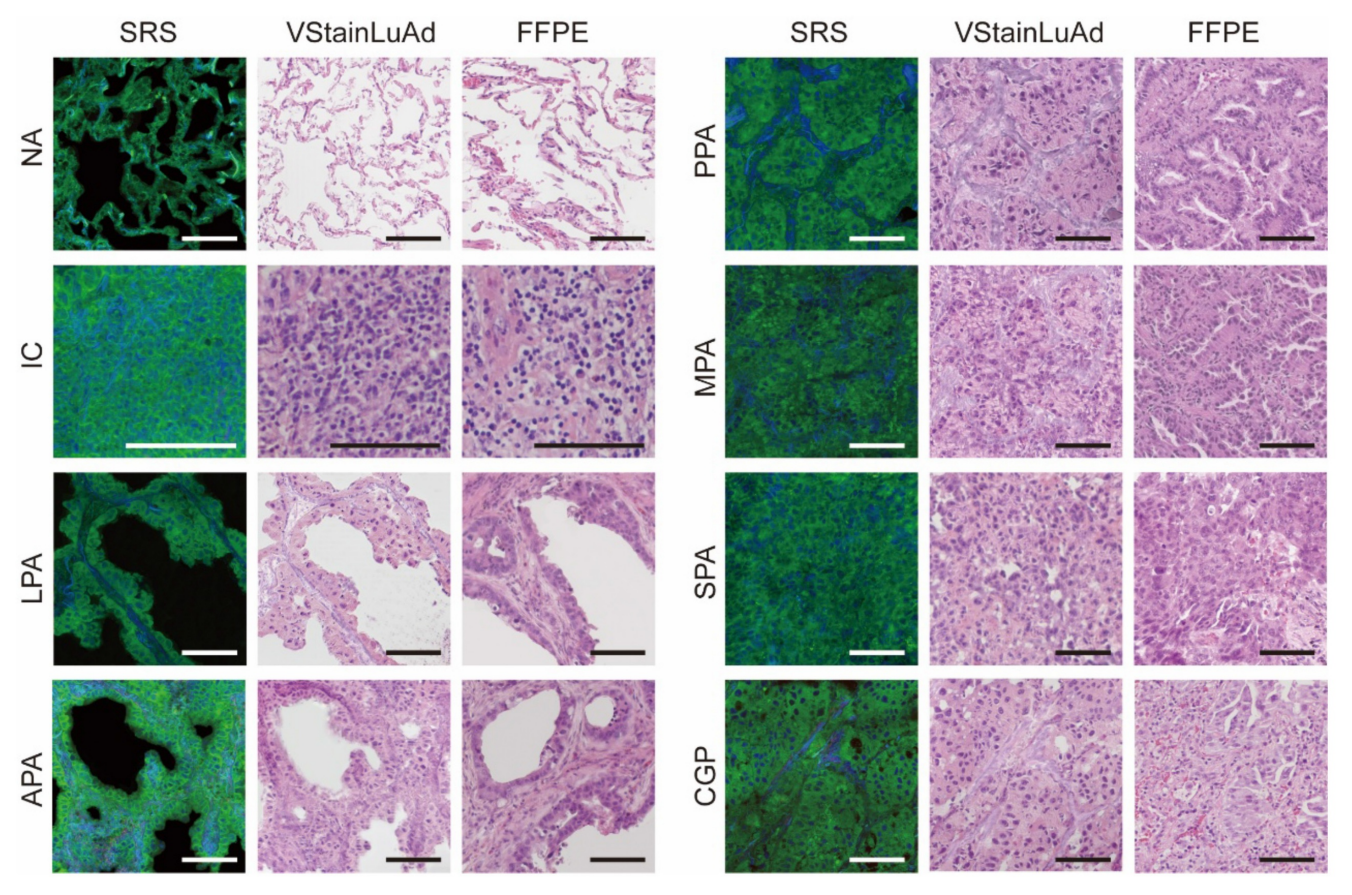
**Representative virtual staining results for eight subtypes of lung tissue.** NA: Normal alveoli; IC: immune cells; LPA: lepidic predominant adenocarcinoma; APA: acinar predominant adenocarcinoma; PPA: papillary predominant adenocarcinoma; MPA: micropapillary predominant adenocarcinoma; SPA: solid predominant adenocarcinoma; CPG: complex glandular patterns. Scale bars: 100 µm.

The robustness and broad applicability of VStainLuAd could be demonstrated in the virtual staining of all representative lung tissue regions and subtypes (Figure 6), including normal alveoli, immune cell aggregates, low-, intermediate-, and high-grade adenocarcinoma subtypes (LPA, APA, PPA, MPA, SPA), and complex glandular patterns (CGP). From left to right, each row shows the original SRS image, the stimulated Raman virtual histology (SRVH) generated by VStainLuAd, and the corresponding real FFPE H&E section. The results show that VStainLuAd successfully reconstructs tissue-specific features consistent with FFPE. For instance, LPA and APA subtypes preserve glandular morphology and exhibit light cytoplasmic staining, while MPA and SPA subtypes demonstrate dense cellularity and deeply stained nuclei—hallmarks of high-grade malignancy. To quantitatively evaluate the fidelity of virtual H&E staining, we implemented a scoring framework to assess artifact prevalence in the generated images. Each 512 × 512-pixel image patch was rated a score on a five-level, 10-point scale, where lower scores indicate severe morphological distortion (2, 4), mid-range scores reflect partial structural preservation with noticeable artifacts (6), and higher scores (8, 10) correspond to minimal artifacts and morphology comparable to clinical FFPE H&E. Two senior pathologists jointly performed blinded evaluation on randomly sampled patches from different model outputs. Using this scoring system, conventional CycleGAN showed a mean score of 6.20, whereas VStainLuAd achieved a significantly higher mean score of 9.66, approaching the score of real FFPE H&E images (9.68), demonstrating that semantic structural guidance effectively improves staining fidelity and reduces artifacts (Figure S9).

To examine whether dataset size contributed to the performance of VStainLuAd, we conducted a dataset-size ablation study by training separate models using 25%, 50%, 75%, 90%, and 100% of the available training data (Figure S9). The scoring results revealed a marked improvement as the dataset fraction increased from 25% to 75%, followed by a performance plateau when using ≥ 90% of the dataset, indicating that our current training dataset lies within the stable and saturating performance regime. These confirm that the training data is sufficient to support reliable virtual staining. Thus, representative FFPE comparison together with blinded pathologist scoring provides appropriate validation of virtual staining fidelity.

Additionally, to evaluate the effectiveness of incorporating brain SRS pre-training in the virtual staining framework, we compared the generator convergence behavior with and without pre-training. As shown in Figure S9, models initialized with brain SRS data exhibited faster convergence and consistently lower final generator loss in both translation directions (SRS→H&E and H&E→SRS), indicating improved training stability and reduced artifacts. This quantitative observation is further supported by the pathologist scoring results (Figure S9), where the pre-trained model achieved a higher average score (+1.33) compared to the non-pre-trained model.

## Methods

### Tissue collection and dataset creation

This research was approved by the Institutional Ethics Committee of Huadong Hospital affiliated with Fudan University with written informed consent (approval no. 20210091). A summary of the patient cohort is presented in Figure S2. Fresh lung tissues excised during surgery were immediately transported to the pathology department. Some of the tissues were sectioned using a freezing microtome (Leica CM1950), generating two adjacent sections of 5 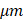
and 10 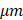
in thickness. The 5
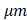
section was immediately subjected to H&E staining, while the 10
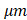
section remained unstained and was promptly transferred to the laboratory under a -20°C frozen environment for SRS-SHG imaging. The dual-channel SRS images were linearly decomposed to obtain lipid and protein distributions, together with the SHG image formed an RGB composite. The corresponding adjacent H&E-stained image was captured using a CCD camera, rescaled to match the resolution of the SRS-SHG images, and stitched into a large-scale image. Subsequently, pathologists referenced the adjacent H&E-stained sections and used the LabelMe software to precisely annotate the SRS-SHG images, facilitating the construction of the segmentation network dataset. It should be noted that pixel-level annotations were performed exclusively on SRS images, while the corresponding H&E sections were used solely as morphological references to guide the annotation process, rather than as direct inputs for model training. Additionally, comprehensive pathological diagnoses of the same patient, obtained through the standard FFPE H&E staining method, were collected as reference data and recorded in the database.

Additionally, during model training, standard data augmentation techniques were applied to enhance generalization and prevent overfitting. Each input image was randomly rotated (±15°), flipped horizontally or vertically, and subjected to mild intensity perturbation and Gaussian noise. These augmentations simulated variations in tissue orientation, brightness, and imaging conditions while maintaining histological integrity.

### Set-up of SRS microscopy

To capture stain-free images, SRS-SHG microscope was employed. A schematic of the setup is shown in Figure S1. The light source consisted of a femtosecond optical parametric oscillator (Insight X3+, Newport), which provided a fixed Stokes beam at 1045 nm (~200 fs) and a tunable pump beam ranging from 680 nm to 1300 nm (~150 fs). The Stokes beam was modulated by an electro-optical modulator at a repetition rate of 20 MHz. Both the pump and Stokes beams were linearly chirped to picosecond durations using SF57 glass rods, ensuring sufficient spectral and chemical resolution. After spatial and temporal overlap, the two laser beams were directed into a laser scanning microscope (FV3000, Olympus) and tightly focused onto the tissue samples using a water immersion objective lens (UPLSAPO 60XWIR, NA 1.2, Olympus). The transmitted stimulated Raman loss signal was optically filtered using a bandpass filter (ET890/220, Chroma) and detected by a custom-built photodiode. The resulting electronic signal was then demodulated via a lock-in amplifier (HF2LI, Zurich Instruments) and fed into the microscope's analog input to generate images. The target Raman frequency was tuned by adjusting the time delay between the two pulses. For histological imaging, data were acquired at two delay positions corresponding to the 2,845 cm⁻¹ and 2,930 cm⁻¹ channels. The raw images were then processed using a numerical algorithm to decompose them into lipid and protein distributions 27, 48, 49, resulting in two-color SRS images. For second harmonic generation (SHG) signal detection, a narrow bandpass filter (FF01-405/10, Semrock), and a photomultiplier tube were used in the epi-detection mode. All images were acquired with 512 × 512 pixels and a pixel dwell time of ~2 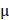
s. The spatial resolution of the system was approximately 350 nm. To capture a larger tissue area, mosaicking and stitching techniques were applied to combine multiple small fields of view into a single, seamless image. The laser powers at the sample were maintained at 50 mW for both the pump and Stokes beams.

### SegLuAd architecture, training procedure, and case-level grading strategy

To achieve accurate recognition and pixel-level segmentation of diverse histological components in lung adenocarcinoma, we developed a semantic segmentation network named SegLuAd, based on the Swin Transformer architecture. A detailed workflow is illustrated in Figure S3. The model was implemented using the MMSegmentation framework and integrates a UPerNet decoder with a Swin-Tiny backbone. The Swin-Tiny backbone consists of four hierarchical stages with 2, 2, 6, 2 transformer blocks, offering strong multiscale feature representation and contextual modeling suitable for structurally complex multimodal pathology images.

SegLuAd was designed specifically for SRS images and outputs 8 semantic classes: normal alveoli, immune cells, low-grade, intermediate-grade, high-grade, stroma, tracheal wall, and background. All input images are multichannel SRS images, representing chemical distributions of lipids, proteins, and collagen within lung tissues.

During preprocessing, each original large image was cropped to a resolution of 2048 × 2048 pixels to ensure sufficient structural context. The cropped images were then uniformly resized to 600×600 and randomly cropped to 512 × 512 before input into the network. Z-score normalization was applied using zero mean and unit standard deviation (mean = [0, 0, 0], std = 1, 1, 1), followed by data augmentation including random horizontal flipping and brightness distortions. Unlike traditional random cropping strategies, we adopted a resize-first approach to retain overall tissue integrity and avoid loss of critical lesion boundaries.

The model was trained using the AdamW optimizer with an initial learning rate of 1×10⁻⁴ and a weight decay of 0.01. A polynomial learning rate decay schedule was applied throughout training. The loss function combined Cross-Entropy Loss and Dice Loss to balance pixel-wise accuracy and sensitivity to small regions.

Training data were organized in the PASCAL VOC12 format. A total of 101 annotated SRS images from 71 lung adenocarcinoma patients were used. The dataset was split into training/validation and test sets in an 8:2 ratio, and 5-fold cross-validation was conducted on the training/validation set to assess model robustness. During inference, test images were resized to 512 × 512, and the output semantic maps were used for quantitative analysis and case-level grading.

To achieve automatic histological grading at the case level, we adopted a rule-based strategy based on the guidelines of the International Association for the Study of Lung Cancer (IASLC). Specifically:

1. For each SRS image, the segmented regions were analyzed to compute the proportion of each histological subtype;

2. Low-grade (lepidic), intermediate-grade (acinar/papillary), and high-grade (solid/micropapillary/complex glands) labels were grouped accordingly;

3. If any high-grade component exceeded 20% of the total tumor area, the case was classified as high-grade;

4. If no high-grade component exceeded the threshold, the dominant subtype by area determined the final grade;

5. The predicted histological grade was then compared with the corresponding pathologist-reported ground truth.

This approach integrates pixel-level segmentation accuracy with expert-defined diagnostic rules, enabling fully automated, reproducible histological grading of lung adenocarcinoma in label-free tissue sections.

Intersection over Union (IoU) and mean IoU (mIoU) are defined as follows:

Let:

- C: total number of classes;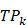: number of true positive pixels for class k;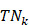: number of true negative pixels for class k;
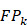
​: number of false positive pixels for class k;
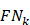
​: number of false negative pixels for class k;

Then the IoU for class k is:


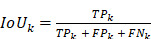
;

Precision is defined as:


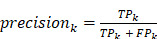
;

Recall for class k is defined as:


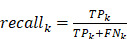
;

Accuracy for class k is defined as:


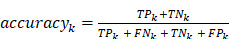
;

Dice coefficient for class k is defined as:


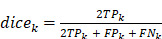
;

To assess the accuracy of boundary localization, we adopted the Boundary F1 (BF1) score, which evaluates how well the predicted segmentation boundary aligns with the ground-truth boundary. We denote by 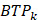
the number of true positive boundary pixels for class k, and similarly for 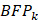, 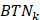
and 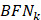. We set tolerance to 6 pixels to account for minor sectioning- and registration-related variations. Then, the Boundary F1 score is defined as:


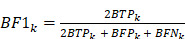
;

### Automated analysis platform

The automated analysis platform is primarily based on our custom-developed Python program, enabling automated quantification of lipid content, protein content, and lipid-to-protein ratio, as well as cytological metrics such as cell density, cell area, and nucleus-to-cytoplasm ratio. All relevant Python scripts have been modularized according to their specific functions and are included as supplementary files for reference.

Lipid and protein content analysis is performed using segmented colormap images, where grayscale lipid and protein images are pixel-wise segmented based on the classified subtypes or tissue types, generating a series of new images for each category. To ensure consistent weighting across all statistical data points, an image tiling algorithm further partitions these images into smaller 512 × 512-pixel patches. Subsequently, the program calculates the average lipid or protein intensity at the patch level by dividing the total intensity of all pixels within the non-black regions by the corresponding pixel count. Naturally, the lipid-to-protein intensity ratio can also be obtained using the same method.

A crucial step in cytological-level quantification is cell identification. Given that the analysis targets nucleated cells and nuclear segmentation is relatively straightforward, we trained a U-Net network to identify cell nuclei. First, using both the segmented colormap images and SRS images, we generated class-specific images containing only low-, intermediate-, or high-grade regions. Since the segmentation step had already separately identified stromal structures, including alveolar walls, these components were excluded from the images, ensuring that only the cells corresponding to each tissue class were retained.

Next, an image tiling module divided these images into smaller 512 × 512-pixel patches. Due to the well-defined nuclear boundaries in the multimodal images, nuclei could be readily annotated using the LabelMe software. A total of 300 randomly selected patches (100 per class) were manually labeled and used to train the U-Net model. Nuclei segmentation was performed using a U-Net architecture with a four-level encoder-decoder design and skip connections to preserve spatial detail. Input SRS images were normalized with data augmentation including random rotation, horizontal/vertical flips, and mild intensity jitter. The model was trained for 200 epochs using the Adam optimizer, and optimized using a combined Cross-Entropy and Dice loss. After training, the network could generate large-scale nuclear segmentation results within seconds (Detailed workflow along with representative output examples are shown in Figure S6).

Subsequently, the nuclear quantification algorithm applied connected-component analysis with a minimum pixel threshold to count nuclei. To maintain consistent weighting across large-scale data, an image tiling algorithm was used to segment large images. To prevent double-counting of nuclei located at image edges, the algorithm assigned a weight of 0.5 to edge nuclei, ensuring accurate quantification. The average cell area was calculated by dividing the non-black region of an image by the total number of nuclei, utilizing the pixel-to-micron conversion factor of 2.8986 pixels/μm. Similarly, the average nucleus-to-cytoplasm ratio and cell density were computed using analogous automated algorithms.

### Virtual staining via VStainLuAd

To achieve virtual H&E staining of SRS images, we constructed an image-to-image translation model named VStainLuAd, based on the unsupervised CycleGAN framework. The detailed workflow is shown in Figure S6. The model consists of a bidirectional architecture with two generators (G₁ and G₂) and two discriminators (D₁ and D₂), which learn to translate between the SRS and H&E image domains using unpaired training data.

Due to the morphological complexity and heterogeneity of lung tissue, conventional CycleGAN models are prone to producing color distortions and structural artifacts during style transfer. To enhance structural awareness, we introduced a semantic guidance mechanism. Specifically, pixel-wise segmentation probability maps were generated from SRS images using the pretrained SegLuAd network. These maps were normalized and fused with the original SRS images via an alpha-masking operation to form structure-aware inputs. Different tissue regions were assigned varying weights to emphasize morphologically informative areas while suppressing less relevant background, thereby improving the fidelity of tissue structure reconstruction.

We adopted a two-stage training strategy. In the first stage, the model was pretrained on brain SRS images, which exhibit relatively consistent lipid and protein distributions, allowing the generator to learn stable base mappings from SRS signals to the H&E domain. In the second stage, the model was fine-tuned on lung SRS images with the integration of semantic guidance to improve generalization in more complex tissue contexts.

All input images were resized to 512 × 512 pixels. We used the Adam optimizer with β₁ = 0.5 and β₂ = 0.999, and an initial learning rate of 2 × 10⁻⁴, followed by linear decay scheduling. This learning rate setting follows common practice in CycleGAN-based image translation tasks and offers a robust balance between image quality and training stability. The full training objective combined adversarial loss, cycle-consistency loss, and identity loss, balanced with weights 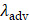
= 1, 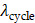
= 10, and 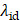
= 0.5. The final trained VStainLuAd model can generate high-fidelity virtual H&E images from raw SRS data without requiring any manual annotations or paired images. These virtually stained images preserve both biochemical contrast and histological morphology, providing a unified visual platform for downstream histopathological analysis.

## Discussion and Conclusions

This study demonstrates that semantic-guided analysis of label-free SRS images enables accurate histologic grading, quantitative biochemical profiling, and high-fidelity virtual staining of lung adenocarcinoma. Leveraging a segmentation-driven pipeline, DeepLuAd provides pixel-level identification of histologic components, class-specific molecular analysis and virtual staining within an integrated framework, offering a scalable alternative to conventional histology for intraoperative diagnosis and research applications.

Previous studies combining SRS microscopy with deep learning have primarily focused on patch-level classification of tissue types or tumor versus normal discrimination. While these approaches can aid rapid screening, they lack the spatial granularity needed to capture heterogeneous histological patterns within individual lesions. In contrast to conventional toolboxes implemented in R, Python, or MATLAB (e.g., EBImage, scikit-image, MATLAB Image Processing Toolbox) for general image analysis, DeepLuAd is particularly tailored for the biochemical contrast and structural complexity of SRS data. This disease-specific, multi-task framework unifies pixel-level segmentation, biochemical quantification, and virtual staining within a single architecture, enabling direct class-specific quantification and downstream analysis for lung adenocarcinoma. Comparative evaluations with established architectures (including U-Net, DeepLabV3, and CycleGAN) demonstrate that DeepLuAd achieves higher segmentation fidelity and virtual-staining consistency, particularly in regions with complex glandular and stromal organization (Table S1 and Figure 5). Our results further revealed a gradual increase in the lipid-to-protein ratio and tumor cell density across low-, intermediate-, and high-grade lesions. These observations are consistent with reported metabolic reprogramming in cancer, where enhanced lipid synthesis and storage support rapid membrane biogenesis, energy supply, and invasive behaviors of aggressive tumor cells 52, 53, 59. By providing a framework for automated extraction of class-specific biochemical and cytological metrics, QuantLuAd enables not only quantitative support for clinical diagnosis but also facilitates fundamental studies on tumor metabolism and progression. Once these quantitative metrics are validated on larger cohorts, they may provide complementary diagnostic markers beyond morphological grading and serve as valuable tools for translational researches.

The semantic guidance strategy also improved the performance of the CycleGAN-based virtual staining module. By incorporating structure-guided semantic priors from the segmentation network, the model produced H&E-like images with better preservation of nuclear and stromal features, reducing the artifacts commonly seen in purely unsupervised image-to-image translation. This highlights the advantage of combining label-free imaging with semantic-guided deep learning for clinically interpretable digital pathology. The current study has several limitations that warrant consideration. First, manual annotations were created by pathologists referencing both SRS and adjacent H&E-stained slices to delineate tissue structures. While this approach ensured high labeling accuracy, it remains labor-intensive and dependent on expert experience, making large-scale dataset construction challenging. Future efforts should explore weakly supervised, semi-automated, or active learning-based annotation strategies to reduce the time and expertise required for high-quality pixel-level labels. Second, our dataset was relatively small and derived from a single medical center, which may restrict generalization across patient populations, imaging devices, or rare histological variants. Future multi-center collaborations with larger dataset and more diverse cohorts will be essential for further validating the robustness and clinical adaptability of the framework. Third, current SRS technology is limited by imaging speed, depth penetration, equipment cost, and the need for trained operators, constraining its immediate adoption in routine pathology workflows. Advances in high-speed large-field SRS imaging and more compact devices could mitigate these barriers. Moreover, although lipid-to-protein ratios and cell density metrics revealed interesting associations with tumor grade, their diagnostic and prognostic value requires further large-scale clinical validation. Finally, the virtual staining model, while improved with semantic guidance, is still based on unpaired image translation and may introduce subtle artifacts. Future work should incorporate uncertainty estimation, paired or semi-paired training strategies, and more diverse datasets to ensure robustness.

Collectively, we have systematically investigated the histologic imaging capability using SRS microscopy, and developed DeepLuAd as an integrated AI platform for semantic-guided tumor grading, quantification and virtual H&E staining. Our findings highlight the potential of DeepLuAd as a rapid, quantitative, and interpretable tool for digital histopathology of lung adenocarcinoma, paving the way for broader clinical translation of SRS imaging in cancer diagnosis and research.

## Supplementary Material

Figure S1, schematic of SRS microscopy; Figure S2. overview of the dataset; Figure S3, workflow of SegLuAd network; Figure S4, Ground-truth segmentation and corresponding error map; Figure S5, representative case of intermediate-grade lung adenocarcinoma; Figure S6, U-Net-based nuclei segmentation; Figure S7, QuantLuAd analysis based on ground truth; Figure S8, training workflow of SegLuAd; Figure S9, evaluation of virtual staining results; Table S1, comparison between SegLuAd and other segmentation models; Table S2, external validation of DeepLuAd predictions; Table S3, Spearman correlation and concordance correlation of SegLuAd; Table S4, case-level statistical analysis of segmentation results.

## Figures and Tables

**Table 1:** Quantitative evaluation of SegLuAd across lung tissue classes.

Tissue class	IoU (%)	Acc (%)	Dice coefficient (%)	Precision (%)	Recall (%)	Boundary F1 (%)
Normal alveoli	84.06	98.8	91.34	92.10	90.59	75.63
Low grade	79.23	96.6	88.41	91.15	85.84	72.53
Intermediate grade	74.64	95.2	85.48	94.05	78.34	68.23
High grade	85.16	96.96	91.99	97.29	87.23	79.25
Stroma	87.94	96.86	93.58	95.76	91.50	83.45
Immune cell	70.97	98.41	83.02	89.17	77.66	62.63
Tracheal wall	82.93	99.09	90.67	93.61	87.89	76.78
Background	78.47	98.82	87.94	90.56	85.45	72.98

## Abbreviations

AI: artificial intelligence
APA: acinar predominant adenocarcinoma
a.u.: arbitrary units
BF1: Boundary F1-score
CCC: concordance correlation coefficient
CGP: complex glandular patterns
CNN: convolutional neural network
FFPE: formalin-fixed paraffin-embedded
H&E: hematoxylin and eosin
IASLC: International Association for the Study of Lung Cancer
IC: immune cells
IoU: intersection over union
LPA: lepidic predominant adenocarcinoma
mIoU: mean intersection over union
MPA: micropapillary predominant adenocarcinoma
NA: normal alveoli
PPA: papillary predominant adenocarcinoma
SHG: second-harmonic generation
SPA: solid predominant adenocarcinoma
SC: Spearman correlation
SRS: stimulated Raman scattering
SRVH: stimulated Raman virtual histology
U-Net: U-shaped convolutional neural network
