# Supplementary material for: DeepLuAd: Semantic-guided virtual histopathology of lung adenocarcinoma via stimulated Raman scattering
Source: Theranostics. 2026 Jan 1;16(5):2324–41. doi: 10.7150/thno.125443 (PMC12712914; doi:10.7150/thno.125443)
Supplement: Supplementary file 1 — Figure S1, schematic of SRS microscopy; Figure S2. overview of the dataset; Figure S3, workflow of SegLuAd network; Figure S4, Ground-truth segmentation and corresponding error map; Figure S5, representative case of intermediate-grade lung adenocarcinoma; Figure S6, U-Net-based nuclei segmentation; Figure S7, QuantLuAd analysis based on ground truth; Figure S8, training workflow of SegLuAd; Figure S9, evaluation of virtual staining results; Table S1, comparison between SegLuAd and other segmentation models; Table S2, external validation of DeepLuAd predictions; Table S3, Spearman correlation and concordance correlation of SegLuAd; Table S4, case-level statistical analysis of segmentation results. [file thnov16p2324s1.pdf]

Supplementary information for

DeepLuAd: Semantic-guided virtual histopathology of lung adenocarcinoma via stimulated Raman scattering

Liyang Ma[2,#], Yuheng Guo[2,#], Min Du[3,#], Yongjun Cai[3,#], Yingjie He[2], Qingjun Meng[2], Zhijie Liu[2], Yichuan Lan[2], Ming Li[1*], Minbiao Ji[2*], Lin Qi[1*]

[1]Huadong Hospital Affiliated to Fudan University, Department of Radiology, 200040 Shanghai, China.

[2]State Key Laboratory of Surface Physics and Department of Physics, Shanghai Key Laboratory of Metasurfaces for Light Manipulation, Endoscopy Center and Endoscopy Research Institute, Zhongshan Hospital, Fudan University, 200433 Shanghai, China.

[3]Huadong Hospital Affiliated to Fudan University, Department of Pathology, 200040 Shanghai, China.

[#]These authors contributed equally: Liyang Ma, Yuheng Guo, Min Du, Yongjun Cai.

[*]Email: minli77@163.com; minbiaoj@fudan.edu.cn; qi_lin@fudan.edu.cn

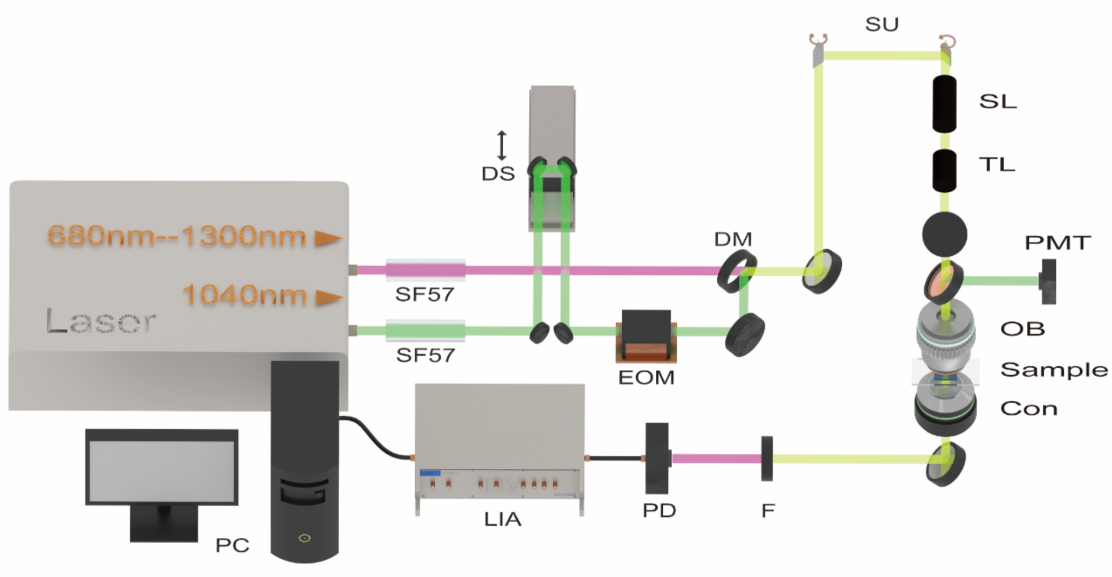

Figure S1. Schematic of the SRS microscopy system. A dual-output femtosecond laser (680–1300 nm tunable pump, 1045 nm fixed Stokes) is used for multimodal imaging. Both beams are chirped using SF57 rods and combined via a delay stage (DS) and dichroic mirror (DM). The Stokes beam is modulated by an electro-optic modulator (EOM) at 20 MHz. The combined beams enter an Olympus FV3000 laser scanning microscope and are focused on the sample through a water immersion objective (OB). Forward-detected stimulated Raman loss signals are collected by a photodiode (PD), filtered (F), and demodulated by a lock-in amplifier (LIA). SHG signals are collected in epi-mode using a photomultiplier tube (PMT). The system acquires label-free histological images at 2845 cm^{-1} and 2930 cm^{-1} with a spatial resolution of ~350 nm.

Abbreviations: PC: personal computer; SF57: SF57 glass rod; DS: delay stage; EOM: electro-optic modulator; DM: dichroic mirror; PD: photodiode; F: filter; LIA: lock-in amplifier; SU: scan unit; SL: scan lens; TL: tube lens; OB: objective lens; Con: condenser lens; PMT: photomultiplier tube.

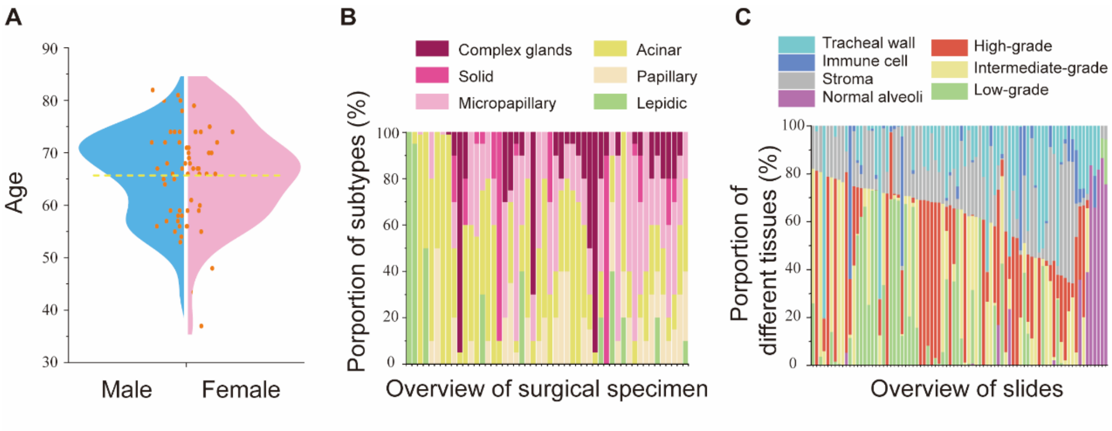

Figure S2. Overview of the dataset used for training and validation. (A) Age distribution of the 50 patients included in the annotated dataset, shown as a gender-separated violin plot with overlaid scatter points. (B) Distribution of lung adenocarcinoma histological subtypes across 50 surgical specimens. Each bar represents one case, with the relative proportion of each subtype displayed. (C) Tissue composition across 80 annotated slides. Stacked bar plots indicate the proportional area of each tissue type identified by pixel-level annotation, including tumor grades and non-tumor components.

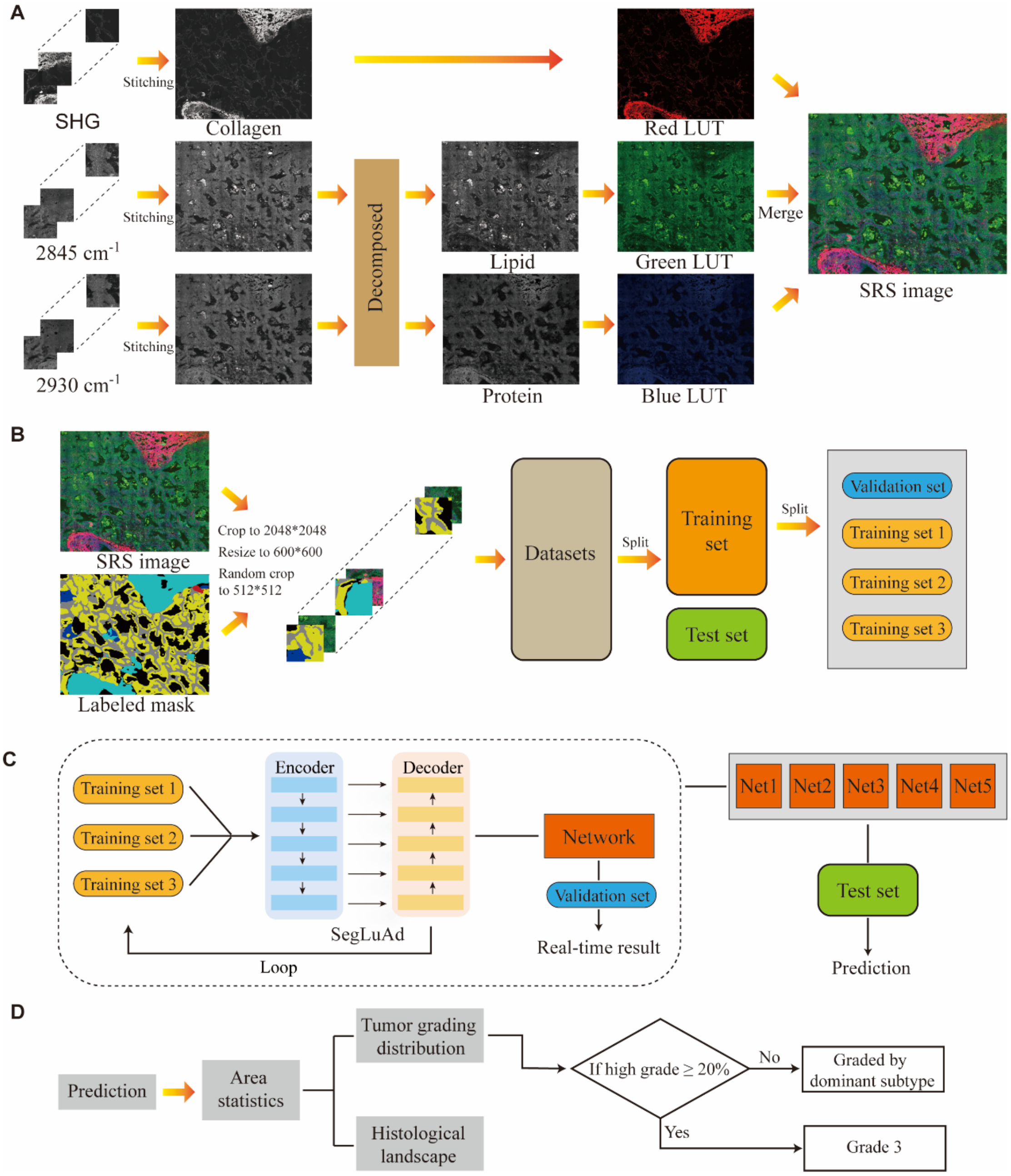

Figure S3. Workflow of SegLuAd training and case-level histological grading. (A) Generation of SRS images. Raw images acquired at 2845 cm^{-1}, 2930 cm^{-1}, and SHG channels are stitched and decomposed into lipid, protein, and collagen components. These are mapped to green, blue, and red channels respectively and merged to form multichannel SRS images. (B) Dataset preparation and preprocessing. Annotated SRS images and corresponding pixel-level labeled masks are cropped to 2048×2048 pixels, resized to 600×600, and randomly cropped to 512×512. The dataset is split into training, validation, and test sets for network development. (C) Training of the SegLuAd network. The model is trained using 5-fold cross-validation on the training/validation set and

evaluated on the independent test set. The architecture includes a Swin Transformer-based encoder and UPerNet decoder. (D) Case-level tumor grading strategy. Following prediction, the proportional area of each histological subtype is computed. If high-grade components exceed 20% of the tumor area, the case is graded as Grade 3; otherwise, the grade is determined by the dominant subtype.

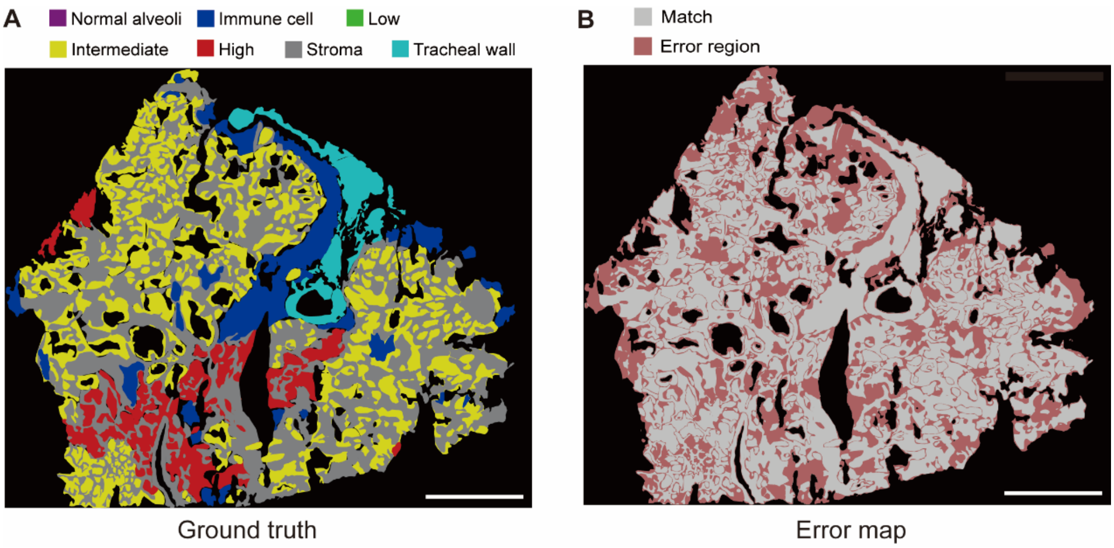

Figure S4. Ground-truth segmentation and corresponding error visualization map. (A) Reference ground-truth annotation of the whole-tissue section. (B) Error map comparing prediction with the ground truth. Gray regions indicate agreement between prediction and annotation, while dusty-rose regions indicate disagreement. The error map highlights local boundaries and subtype-transition zones where misclassification most frequently occurs. Scale bars, 1000 μm.

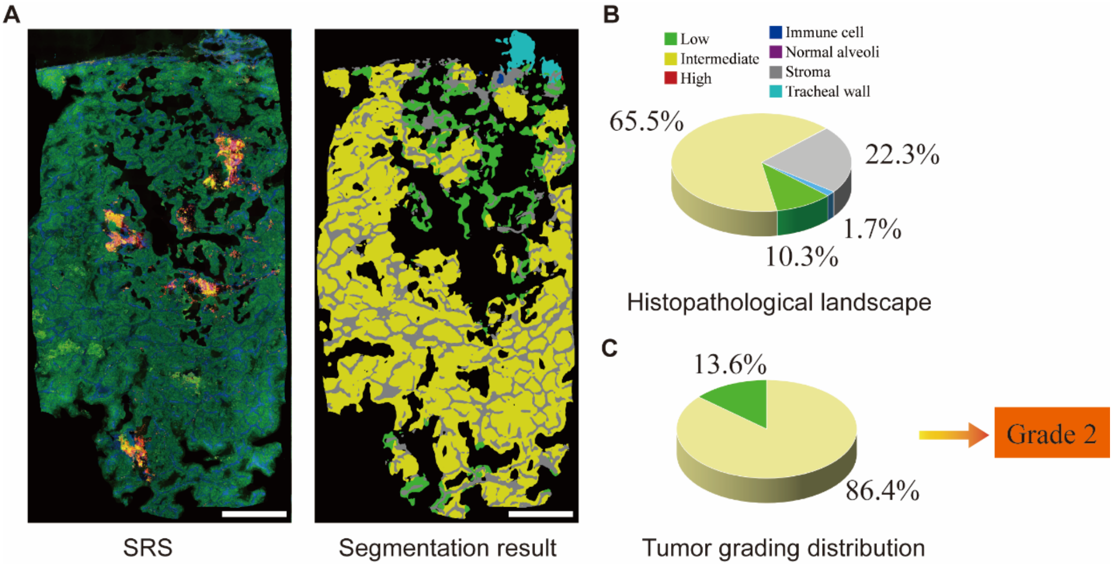

Figure S5. Representative case of intermediate-grade lung adenocarcinoma analyzed by SegLuAd. (A) Left: SRS image. Right: Corresponding pixel-level segmentation result generated by SegLuAd, delineating different histological components. Scale bar: 1000 μm. (B) Pie chart summarizing the histopathological landscape, with intermediate-grade components (yellow) occupying the largest proportion (65.5%), followed by stroma (22.3%) and other tissue types. (C) Grading distribution based on subtype area proportions. As high-grade components do not exceed 20% of the total tumor area, the case is classified as Grade 2, determined by the dominant intermediate-grade subtype.

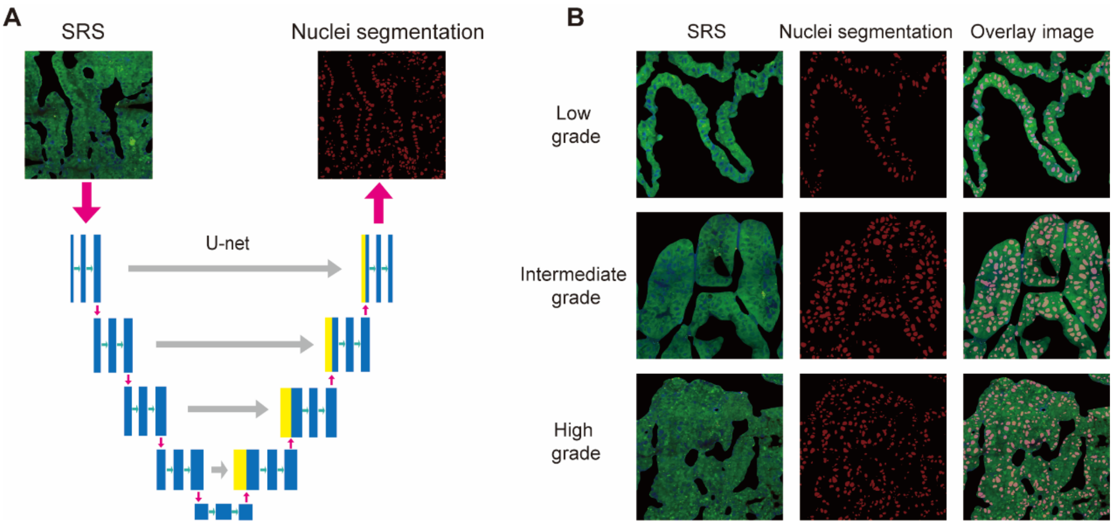

Figure S6. U-Net–based nuclei segmentation from SRS images across different tumor grades. (A) Schematic overview of the U-Net architecture for nuclei segmentation. The multichannel SRS image (input) is processed through an encoder–decoder network to generate pixel-level nuclei segmentation masks. (B) Representative results across low-grade, intermediate-grade, and high-grade lung adenocarcinoma regions. Columns show: original SRS image (left), corresponding nuclei segmentation result (middle), and overlay of segmentation mask on SRS image (right). Nuclei appear in red, accurately delineated regardless of tissue grade or cell density.

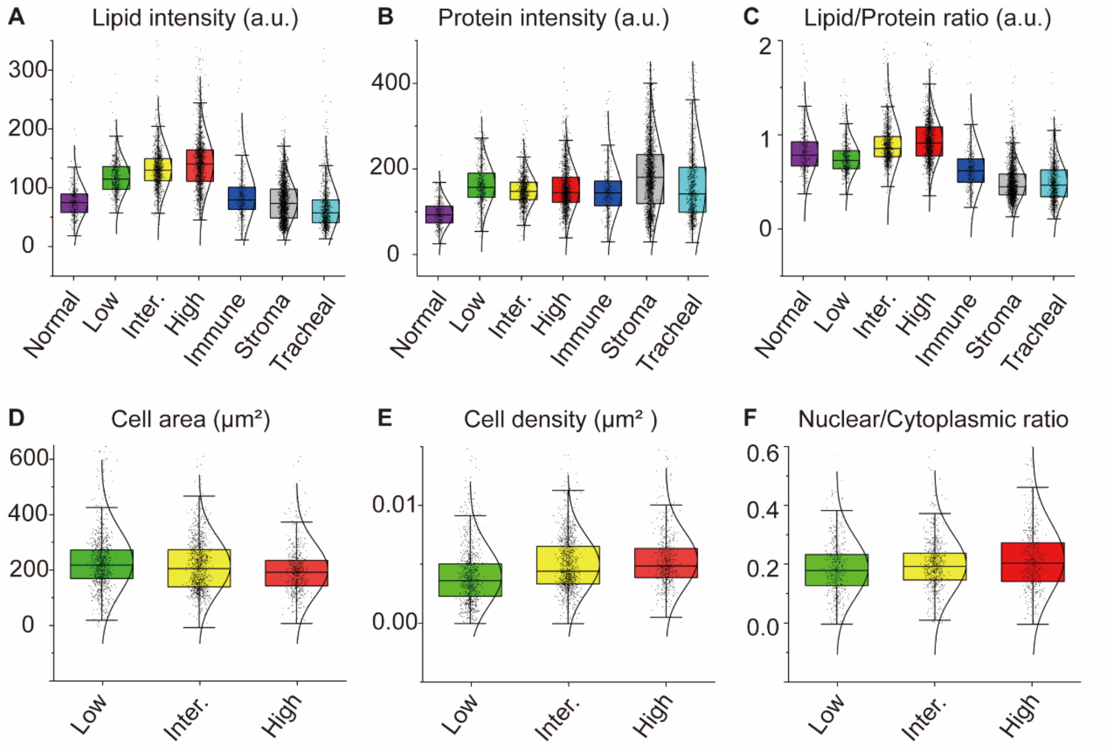

Figure S7. QuantLuAd module for biochemical and cytological analysis based on ground truth. (A-C) Biochemical profiling including the intensity distributions of lipid, protein and lipid/protein ratio across the seven tissue subtypes. (D-F) Cytological profiling including cell size, cell density and nuclear/cytoplasmic ratio in low-, intermediate-, and high-grade tumor regions. a.u.: arbitrary units.

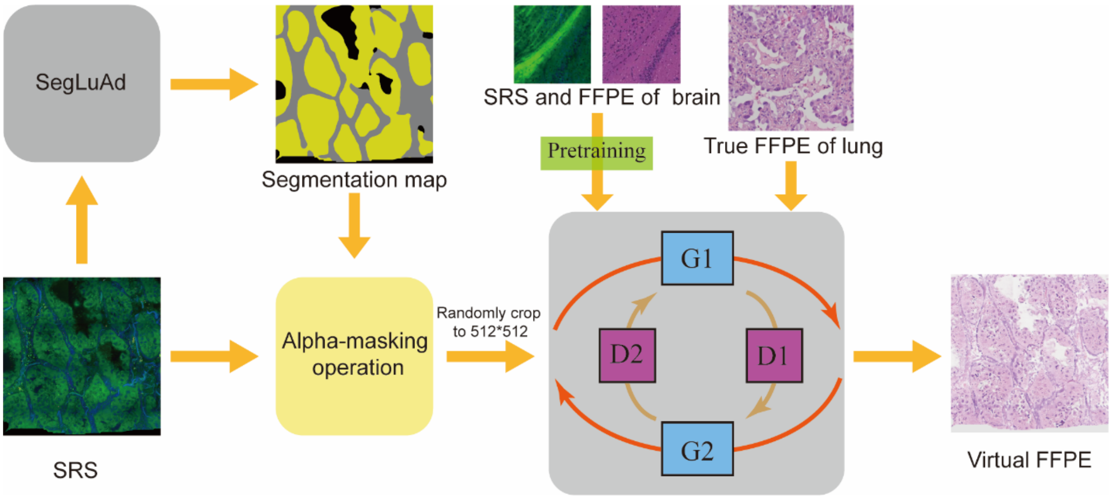

Figure S8. Training workflow of the SegLuAd virtual staining framework. To enable virtual H&E staining from unlabeled SRS images, SegLuAd is trained using an unsupervised CycleGAN architecture. The input consists of multichannel SRS images fused with structure-aware segmentation maps generated by the SegLuAd network. This fusion is performed via alpha masking to emphasize morphologically informative regions. The resulting structure-guided images are randomly cropped to 512×512 patches and fed into a dual-generator, dual-discriminator CycleGAN model (G_1/G_2 and D_1/D_2). In the first stage, the model is pretrained on brain tissue data to learn stable SRS-to-H&E mappings. In the second stage, it is fine-tuned on lung SRS images with semantic guidance to improve staining fidelity in morphologically complex tissue. Training is conducted using unpaired data without manual annotations, with an initial learning rate of 2×10^{-4} and linear decay scheduling.

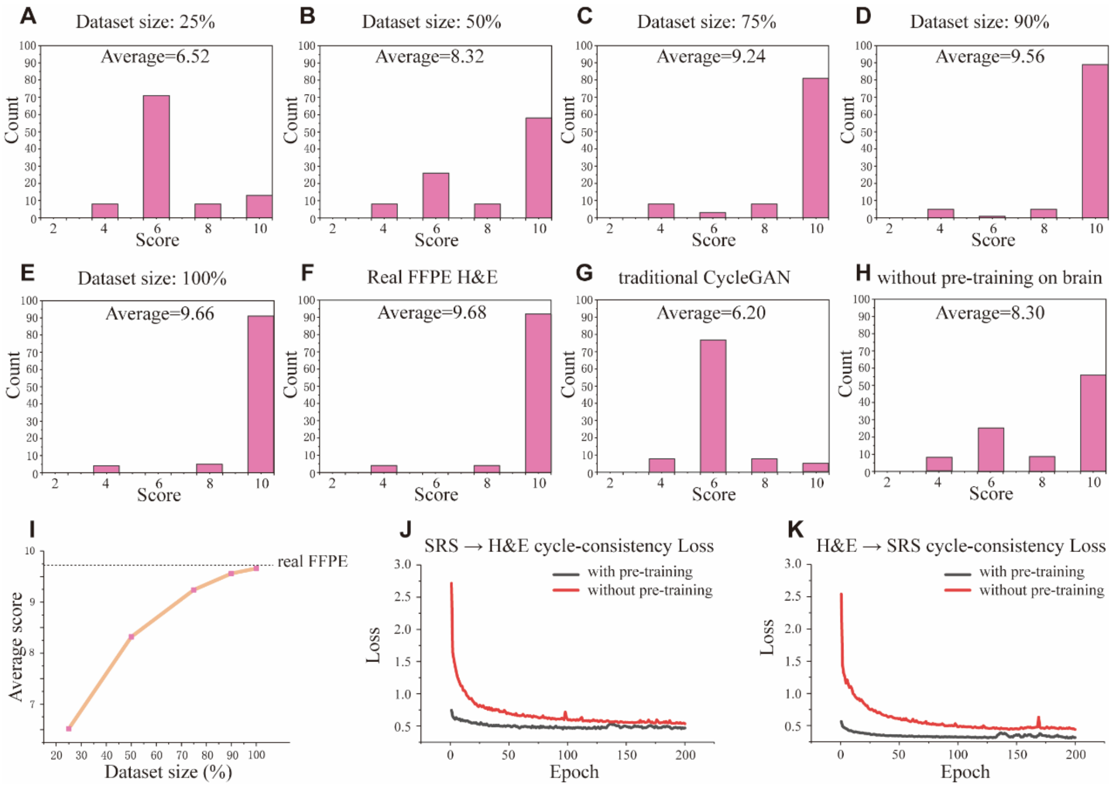

Figure S9. Quantitative evaluation of virtual H&E staining quality, dataset-size dependence, and effect of brain SRS pre-training.

(A–E) Virtual staining quality as a function of training set size. Models were trained with 25%, 50%, 75%, 90%, and 100% of the available FFPE H&E training patches and then evaluated on 100 randomly sampled 512×512 virtual H&E patches per condition. (F) Real FFPE H&E reference. (G) Conventional CycleGAN (no semantic guidance). (H) VStainLuAd trained without brain SRS pre-training. Panel titles report the average pathologist score for each condition (A: 6.52; B: 8.32; C: 9.24; D: 9.56; E: 9.66; F: 9.68; G: 6.20; H: 8.30). (I) Dataset-size vs. quality curve computed from panels A–E, with the dashed line indicating the average score of real FFPE (panel F), showing saturation as training data approach 100%. (J) Cycle-consistency loss curves for the SRS→H&E generator with (black) and without (red) pre-training on brain SRS data. (K) Cycle-consistency loss curves for the H&E→SRS generator under the same conditions. In both translation directions (J and K), models initialized with brain SRS pre-training exhibit faster convergence and lower final cycle-consistency loss,

indicating improved training stability and more reliable structure-preserving translation behavior.

Scoring protocol. Two senior thoracic pathologists jointly established and applied a five-level, 10-point scoring rubric (higher indicates better histological fidelity). The criteria were as follows: 2 = severe artifacts with unreliable morphology; 4 = noticeable artifacts with partial but inconsistent structural preservation; 6 = mild artifacts with overall recognizable tissue morphology; 8 = minimal artifacts and generally reliable histological structures; 10 = no visible artifacts and morphology comparable to standard diagnostic H&E. For each model condition, 100 randomly sampled 512×512 patches were evaluated, and the final score for that condition represents the average rating determined by the two pathologists after consensus discussion.

Table S1. Performance comparison of SegLuAd with other widely used segmentation models. The table summarizes the mean Intersection over Union (mIoU) and mean precision achieved by several representative semantic segmentation networks on the SRS dataset. SegLuAd outperforms all baseline models, including CNN-based (U-Net, PSPNet, DeepLabV3, HRNet) and Transformer-based (ViT, Swin Transformer) architectures. The training of SegLuAd adopts AdamW optimization and incorporates semantic priors from SRS images.

Network model	mIoU (%)	Precision (%)	Optimization method
PSPNet	29.35	43.77	SGD
HRNet	33.38	48.99	SGD
DeeplabV3	36.09	48.33	SGD
U-Net	43.05	59.68	SGD
Vit	72.49	78.61	AdamW
Swin transformer	74.38	85.15	AdamW
SegLuAd (ours)	80.42	92.96	AdamW

Table S2. External validation of DeepLuAd grading predictions across 21 independent lung adenocarcinoma cases. The table summarizes histological subtype proportions and grading results derived from hospital pathology reports (columns 2–5) and DeepLuAd predictions (columns 6–9). Grading follows the IASLC guideline, where cases with >20% high-grade components are classified as Grade 3. Consistency is defined as a match between the predicted and reported grade: C (consistent) or I (inconsistent). DeepLuAd achieved a grading accuracy of 76.2% (16/21).

	Pathological report				Prediction results of DeepLuAd				Consis-tency
	Low (%)	Inter-mediate (%)	High (%)	Grading	Low (%)	Inter-mediate (%)	High (%)	Grading	
#1	70	25	5	1	75.5	24.5	0.0	1	C
#2		60	40	3	0.5	12.8	86.7	3	C
#3	5	65	30	3	0.0	82.3	17.7	2	I
#4		90	10	2	0.5	98.5	1.0	2	C
#5		70	30	3	0.0	68.6	31.4	3	C
#6		95	5	2	0.0	100.0	0.0	2	C
#7		99	1	2	0.0	100.0	0.0	2	C
#8	5	95		2	0.0	65.3	34.7	3	I
#9		30	70	3	0.0	24.7	75.3	3	C
#10		95	5	2	53.4	1.6	45.0	3	I
#11		40	60	3	0.0	0.9	99.1	3	C
#12	60	40		1	96.5	3.1	0.3	1	C
#13	60	40		1	51.7	47.6	0.7	1	C
#14		100		2	8.1	91.9	0.0	2	C
#15	55	45		1	46.6	53.4	0.0	2	I
#16	60	40		1	84.8	15.2	0.0	1	C
#17	10	70	20	2	15.0	85.0	0.1	2	C

#18	60	40	3	0.4	55.2	44.3	3	C
#19	50	50	3	0.1	0.0	99.9	3	C
#20	75	25	3	12.4	77.3	10.3	2	I
#21	55	45	3	2.1	42.8	55.1	3	C

Table S3. Spearman correlation (SC) and concordance correlation coefficient (CCC) values comparing the segmentation results of SegLuAd and Ground Truth. This table presents the statistical agreement between SegLuAd's predictions and the expert annotations (Ground Truth). Higher values in both Spearman and CCC indicate strong consistency between the model's segmentation output and the Ground Truth.

	lipid		protein		lipid/protein		cell area		cell density		nuclear/cytoplasmic	
	sc	ccc	sc	ccc	sc	ccc	sc	ccc	sc	ccc	sc	ccc
Normal aveoli	0.982	0.981	0.980	0.991	0.997	0.997						
Low	0.986	0.981	0.996	0.981	0.984	0.985	1.000	1.000	1.000	0.999	1.000	1.000
Intermediate	0.983	0.997	0.997	0.988	0.991	0.990	0.999	0.992	0.999	1.000	0.999	0.999
High	0.984	0.985	0.992	0.988	0.986	0.995	1.000	0.999	1.000	1.000	1.000	0.999
Stroma	0.994	0.985	0.997	0.993	0.989	0.981						
Immune cell	0.988	0.984	0.984	0.982	0.993	0.983						
Tracheal wall	0.989	0.988	0.988	0.984	0.990	0.998						

Table S4. Case-level statistical analysis of segmentation results based on Ground truth and SegLuAd outputs. The table summarizes the data from individual cases, comparing the sexgmentation results between the manually annotated ground truth and the predictions made by SegLuAd across three tumor grades.

	Based on SegLuAd						Based on Gound truth					
	Lipid			Protein			Lipid			Protein		
	Low-	Inter-	High-	Low-	Inter-	High-	Low-	Inter-	High-	Low-	Inter-	High-
#A	127.4	133.6		171	145.2		123.2	135.0		171.1	144.0	
#B		115.4	143.7		178.4	145		115.4	144.2		178.4	143.0
#C		121.4			163.4			122.5			162.6	
#D		112.7	144.6		167.8	172.6		109.1	144.8		166.2	177.1
#E		121.6	136		159.6	140.3		124.3	135.1		162.1	134.7
#F	120.3	127.9		167.9	165.9		116.2	128.2		168.3	161.5	
#G		130.5	131.8		171	141.2		131.7	130.1		171.6	136.0
#H		138	136.1		148.1	139.9		139.5	138.8		147.4	142.6
#I		134.1	133.5		181.9	163.5		135.6	130.7		181.4	167.2
#J		124			147.2			123.9			149.8	
#K	92.7	123.2		154.9	177		90.4	128.1		155.7	179.5	
#L		135.9	119.7		178.9	139.2		138.2	118.9		182.7	142.1
#M	121	115.9		164.8	170.5		122.7	116.8		167.9	163.7	
#N		133.4	155		174.1	152.9		135.9	156.2		171.3	155.1
#O		144.9	153.7	142	151.8	161.2		150.5	154.6	142.7	146.6	156.2
#P		144			158.3			145.4			159.0	
#Q		123.2	129.8		162.6	176.2		120.1	125.6		166.7	180.3
#R			132.2			174			131.2			175.9
#S	96	111.9		163.9	156.7		99.3	114.3		165.6	161.6	
#T		129.3	129.7		163.1	159.7		129.7	133.7		167.5	154.6
